# Hawk owl irruptions: spatial and temporal variation in rodent abundance drive push and pull dynamics

**DOI:** 10.1007/s00442-022-05283-9

**Published:** 2022-11-19

**Authors:** Svein Dale, Geir A. Sonerud

**Affiliations:** Faculty of Environmental Sciences and Natural Resource Management, Norwegian University of Life Sciences, P.O. Box 5003, NO-1432 Ås, Norway

**Keywords:** Bank vole, Habitat selection, Migration, Rodent cycles, Wood mouse

## Abstract

Bird irruptions are thought to be triggered by low food availability in breeding areas, thereby causing emigration (push factor). However, few studies have tested whether emigrating individuals are drawn towards areas of high food availability (pull factor). The Northern hawk owl (*Surnia ulula*), a rodent specialist, occurs irruptively to southern parts of Fennoscandia. We analysed whether irruption size during 1980–2020 in southeastern Norway was related to rodent abundance at four sites 450–990 km to the north-northeast (potential source areas) and at two sites in southeastern Norway to test push and pull dynamics of irruptions. Irruptions occurred when rodent abundance in potential source areas were low, supporting the push hypothesis. High rodent abundance in potential source areas 1–2 years before irruptions suggested that irruptions were preceded by high reproduction. Upon arrival to southeastern Norway, hawk owls did not encounter high rodent abundance in their main habitat (boreal forest). However, hawk owls stayed in boreal forest in hills in years with higher microtine rodent abundance, but occurred in farmland areas in the lowlands when microtine rodents were less abundant. Use of lowlands coincided with higher than median numbers of wood mouse (*Apodemus sylvaticus*) for 87% of the hawk owls settling in the lowlands, thus suggesting support for the pull hypothesis. In conclusion, hawk owl irruptions to southern Fennoscandia were triggered by low food availability in northern areas (push factor), and appeared to be drawn by high food availability in southeastern Norway to some degree (pull factor).

**Supplementary Information:**

The online version contains supplementary material available at 10.1007/s00442-022-05283-9.

## Introduction

Boreal forests typically show cycles in availability of food for birds, in particular small rodent prey and seeds from masting trees, and bird irruptions are generally thought to occur when food availability declines after a peak breeding season (Lack 1954; Svärdson 1957; Bock and Lepthien 1976; Koenig and Knops 2001; Newton 2006, 2008). Lack of food, possibly in combination with stronger intraspecific competition, may then force individuals to emigrate from the regular distribution area. However, little is known about the factors determining where emigrating individuals go and the conditions they experience in the destination areas. If conditions are predictably negatively correlated between source and destination areas, irruptive birds may be drawn to areas with better conditions than where they originate. Synchrony in masting years of trees and peak rodent years may range over hundreds or even thousands of kilometres (Koenig and Knops 1998; Sundell et al. 2004; Gallego Zamorano et al. 2018; Fay et al. 2020; Selås et al. 2021). Irruptive birds may therefore need to travel long distances to find areas with higher food availability than where they came from, i.e. in areas that are asynchronous with the source areas with respect to fluctuations in food abundance (Newton 2006, 2008; LaMontagne et al. 2020).

The possibility that bird irruptions may be both triggered by low food availability and drawn towards higher food availability fits the concept of push and pull factors (Loe et al. 2009; Byrne et al. 2019). Strong et al. (2015) found that pine siskin (*Spinus pinus*) irruptions were influenced by both push and pull factors. West–east irruptions in North America were triggered by low food availability (push factor), and drawn towards areas of high food availability on opposite sides of the continent (pull factor). Climatic dipoles were assumed to create predictably opposite conditions (Zuckerberg et al. 2020), thereby benefitting siskin individuals moving long distances. There is otherwise a lack of knowledge of the role of pull factors, and hence also a lack in our understanding of the relative roles of push and pull factors, in shaping bird irruptions.

Here we test the push and pull hypotheses for bird irruptions using the diurnal and rodent-specialized Northern hawk owl (*Surnia ulula*, hereafter hawk owl or owl) as a model species. The hawk owl has a Holarctic distribution in the northern boreal forests, where it breeds in peak rodent years (Mikkola 1983; Cramp 1985; Sonerud 1997). The owls emigrate when rodent abundances decline, and may then occur irruptively to areas south of the regular range (Mikkola 1983; Cramp 1985; Newton 2006). These irruptions are well documented through observations by the public, because the hawk owl is diurnal, locates prey from elevated perches like tree tops in open forest or in forest clear-cuts, and is thus quite easy to spot, and also quite tame (Sonerud 1992, 1997; Dale 2017). However, nothing is known about how prey availability affects to which areas hawk owls migrate. Furthermore, habitat selection may influence prey availability if rodent dynamics in different habitats are uncorrelated, but large-scale habitat selection of hawk owls during irruptions has not previously been analysed in relation to rodent abundance.

Hawk owls are scarce breeders in southern Norway (Sonerud 1994), but have occurred in large numbers in autumn and winter in certain years (Sonerud 1994; Dale 2017, 2022). Although hawk owl irruptions have sometimes been claimed to come from Russia (Cramp 1985), analyses have indicated that a northern Fennoscandian origin is more likely, at least during the irruptions investigated in detail (Hagen 1956; Dale 2017). Dispersal distances of the few hawk owls ringed as nestlings in Norway ranged 193–1875 km (mean = 513 km, *n* = 5), and one adult male dispersed 826 km towards northeast after having nested successfully (Sonerud 1994; Bakken et al. 2006). During an irruption in 2016–17, hawk owls were recorded in a wide variety of places, but estimates indicated that most were in boreal forest (Dale 2017). However, hawk owls also appeared at coastal sites and in farmland areas in the lowlands, outside boreal forest (Dale 2017). Because hawk owls normally inhabit northern boreal forests, one may speculate that owls moving into farmland areas in the lowlands do so because of a lack of prey in the boreal forests. If local rodent dynamics in forest and farmland are uncorrelated, the owls may potentially encounter higher prey availability by shifting habitat from forest to farmland.

We summarized information about the magnitude of hawk owl irruptions to the Oslo and Akershus regions of southeastern Norway, an area of ca. 5,400 km^2^, during 1980–2020. Based on 248 observations of hawk owls in this region and rodent abundance indices from six geographically separated areas [four northern sites potentially representing source areas, and two local sites in southeastern Norway (one in boreal forest in the hills and one in the lowlands) representing the destination area], we tested (1) whether irruptions were triggered by low prey availability in northern source areas (push hypothesis), and preceded by potentially good breeding conditions, (2) whether irruptions coincided with high prey availability in southern destination areas (pull hypothesis), and (3) whether habitat selection in destination areas made hawk owls able to fine-tune the location of their home ranges to local differences in prey availability. Regarding the latter question, we specifically tested whether hawk owls occurred in boreal forest at higher elevation as long as there was high prey abundance, and whether they left boreal forest and entered farmland areas in the lowlands when prey abundance was low in boreal forest, and thereby encountered higher food availability in the lowlands.

## Materials and methods

### Study area in southeastern Norway

**Fig. 1:**
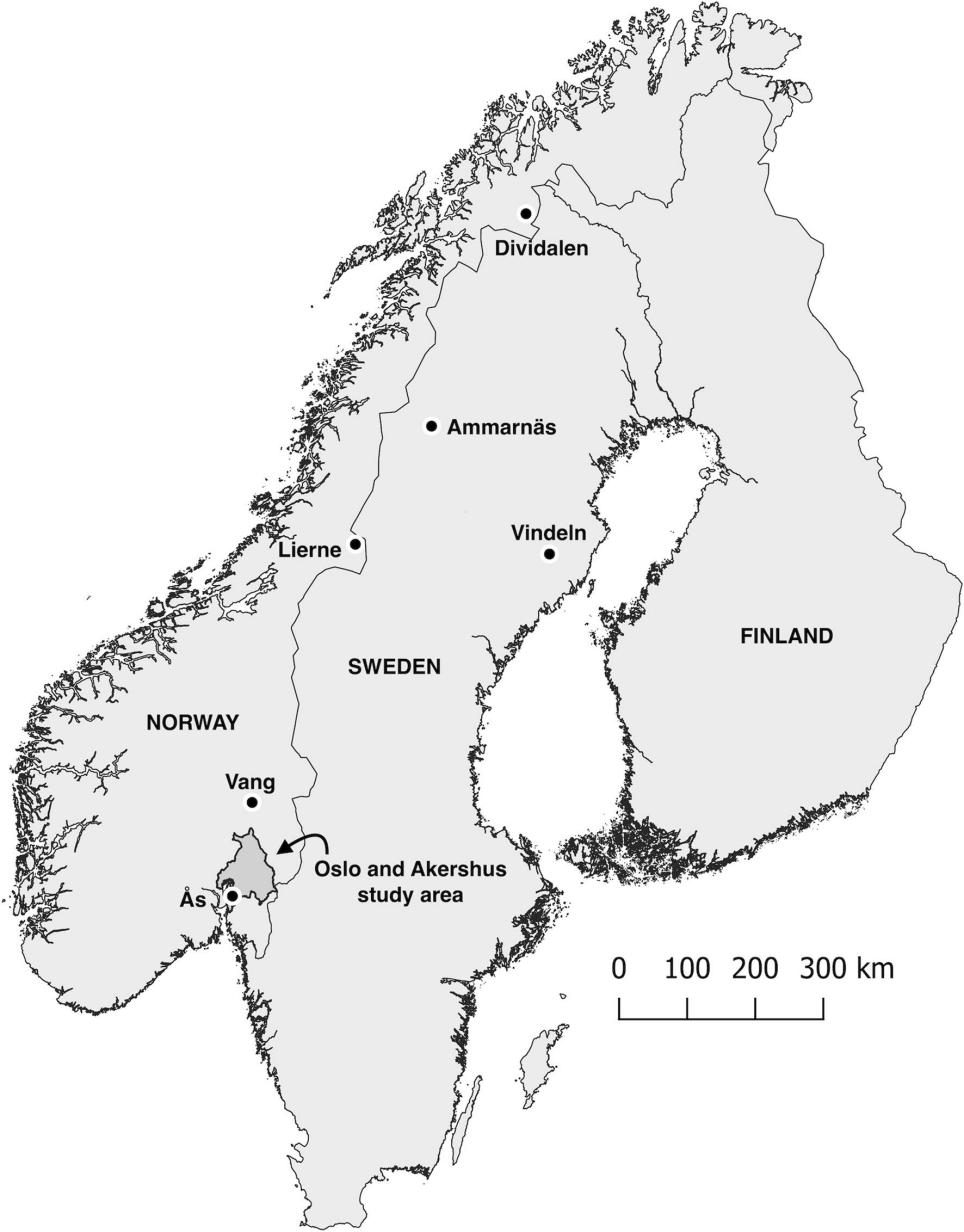
Location of study area (Oslo and Akershus, southeastern Norway, shaded area) and six rodent trapping sites in Norway and Sweden

Data on hawk owl irruptions were collected from Oslo and Akershus counties in southeastern Norway (5372 km^2^ at 59.71–60.46° N, 10.59–11.91° E; Fig. 1). Oslo and Akershus have mostly boreal forests in hills above 200 m a.s.l. The boreal forests are dominated by Norway spruce (*Picea abies*) and Scots pine (*Pinus sylvestris*), and are heavily influenced by modern forestry with a large proportion of clear-cuts. The lowlands (below ca. 200 m a.s.l.) have a large proportion of farmland which is often mixed with forest patches and forest corridors. Southern parts of the lowlands are within the boreo-nemoral zone, otherwise the study area is within the boreal zone with cold winters and regular snow cover. The study area is suitable for the present study because (1) there are many birders who have provided field observations of hawk owls (the local branch of BirdLife Norway has ca. 3,000 member out of a total of ca 11,000 members in BirdLife Norway), and (2) there is knowledge of long-term occurrence of hawk owls (Dale et al. 2001), and (3) the study area has a mix of boreal forest and farmland (65% forest, 14% farmland) whereas the other counties in southeastern Norway most attractive to hawk owls, in particular Innlandet county (Dale 2017), have much less farmland (4% farmland in Innlandet county).

### Irruptions

To obtain data on the number of hawk owls observed in each year, all hawk owl reports were extracted from the websites of the National Biodiversity Information Centre (www.artsobservasjoner.no) and the Oslo and Akershus branch of BirdLife Norway (www.nofoa.no). These websites are online portals for reporting observations of species, and they are open to the public. Most reports of birds are submitted by members of BirdLife Norway. The archives of the Local Reports and Rarities Committee of the Oslo and Akershus branch of BirdLife Norway were also accessed. Due to automatic transfer of reports from www.nofoa.no to www.artsobservasjoner.no, most reports from 2006 to 2021 were also available on the latter website. To remove multiple reports of the same owl individuals, the map plotting function on www.artsobservasjoner.no was used to count the number of different sites (> 2 km distance between plots, following Dale 2017). Two hawk owl females radio-tracked near our rodent trapping site in Vang (see below) had minimum convex polygon home ranges of 2.6 and 4.2 km^2^, respectively, in September–February (Bækken et al. 1987). The distance threshold of 2 km was disregarded if birds had been observed at two close sites simultaneously. Although many hawk owls were reported over extended time periods at some sites, indicating that they may have established a wintering territory, other sites may have been used only for temporary stop-over, or observations may have been of individuals on migration. However, the likelihood that the same individuals had been observed in different sites was considered low.

The number of hawk owl records per year may have been affected by temporal changes in observation effort or reporting frequency. Although the hawk owl is a species attracting the attention of birders so that observations likely have become known to the Local Reports and Rarities Committee, the number of birders has increased over time and web-based bird reporting may have increased reporting frequency over time. To address this issue, we also used (1) hawk owl numbers corrected for observation effort, and (2) a shorter time period (2011–2020, covering three major irruptions) where observation effort changed less. Yearly observation effort was measured as the total number of bird reports submitted each year to the websites mentioned above. However, analyses based on these data showed qualitatively similar results (Online Resource 1, Tables S1–4). Thus, the main text reports the results of analyses based on actual number of known hawk owl records for the whole study period.

Overall, there were 290 hawk owl records during 1980–2021, but 42 records from the breeding season (March–July) were excluded from analyses because breeding may occur in higher-elevation boreal forest in southeastern Norway (Haftorn 1971; Sonerud 1994, 1997). Thus, for each year, the number of hawk owls included records from August–December and records from January–February the following year. For example, 2020 included records from the period August 2020–February 2021, and years therefore refer to when irruptions started. Data were collected for the period August 1980–February 2021, and the data set included 248 records. The data set used in the present study appeared to be representative for a larger area in southeastern Norway because the number of records in Oslo and Akershus was strongly correlated with numbers recorded further south in southeastern Norway (number of records from Østfold, Buskerud, Vestfold, Telemark and Agder counties during 2000–2021, data taken from www.arstobservasjoner.no, where there are fewer reports during the first half of the study period (i.e. before 2000); *r* = 0.89, *n* = 21 years, *P* < 0.001; all peak years were identical), indicating that there was extensive spatial synchrony of owl irruptions in southern Norway. To test whether there was spatial synchrony also within our study area, we divided the study area into two parts. The northwestern part (*n* = 140 records) consisted of the muncipalities Asker, Bærum and Oslo, and areas to the west of the rivers Glomma and Vorma between Lillestrøm and Mjøsa, whereas the southeastern part (*n* = 100 records) consisted of remaining areas of Oslo and Akershus counties. Eight owl records from 1983 had unknown location.

### Elevation and habitat

Elevation of sites with records of hawk owls was measured from www.norgeskart.no. For sites with multiple records within a distance of 2 km, the elevation used was either based on the most used position or the first of positions used equally often. The habitat of records was classified as forest or farmland. Classification was based on the dominant habitat within a radius of 1 km around records, not the habitat of the exact geographical position of the record. Thus, most areas above 200 m a.s.l. were classified as forest, whereas observations from the lowlands were generally in farmland unless the owl was in a large forest patch. The few observations that were from urban areas and other human-dominated places were included in farmland. Because forest habitat was in general at higher elevation, and farmland in the lowlands, the variables elevation and habitat are two alternative measures of the large-scale spatial distribution of the hawk owls. Out of the 248 records used, 233 provided data on elevation and 237 provided data on habitat. Of records below 220 m a.s.l., 25% (31 out of 124) were classified as having forest habitat, whereas of records at or above 220 m a.s.l., 2% (2 out of 109) were classified as having farmland habitat. Because the diurnal hawk owls locate prey from elevated perches like tree tops in open forest or in forest clear-cuts in boreal forest we regarded detectability in boreal forest to be fairly high, although some individuals may have occurred inside more dense forest. Detectability in more open farmland habitat in the lowlands may in general be assumed to be higher than in boreal forest, but the farmland habitats in the study area had a high proportion of forest patches so some hawk owls may remain undetected in this habitat too. Thus, we did not adjust numbers observed in relation to habitat.

### Rodent abundance

Indices of rodent abundance were from six geographically separated areas (Fig. 1); four sites in potential source areas > 400 km north-northeast of the main study area (Lierne in Trøndelag county in Norway, Vindeln in Västerbotten in Sweden, Ammarnäs in Lappland in Sweden, and Dividalen in Troms and Finnmark county in Norway) and two sites in or close to the main study area in southeastern Norway (Vang in Innlandet county, Ås in Akershus county). Data from sites in potential source areas were taken from Sørensen et al. (2020: Lierne), Ecke and Hörnfeldt (2021: Vindeln and Ammarnäs), Kålås et al. (1994: Dividalen) and Framstad (2021: Dividalen). Data from Vang and Ås were collected by one of the authors (GAS).

In southern Norway, breeding hawk owls switch prey from mostly bank voles (*Myodes glareolus*) as long as the ground is snow-covered to mostly field voles (*Microtus agrestis*) or tundra voles *(Microtus oeconomus*) when the snow disappears in spring (Sonerud 1986; Nybo and Sonerud 1990). Bank vole may therefore be an important prey for irrupting hawk owls in autumn and winter. Hawk owls in Norway and elsewhere in Fennoscandia include all microtine species in the diet (Hagen 1952; Ims 1982; Mikkola 1983; Bohlin 1985; Cramp 1985), although the frequency depends on the local availability (Sonerud 1986; Nybo and Sonerud 1990). Murids, including *Apodemus* spp., have been documented as prey, but in low numbers (Mikkola 1971).

At Lierne, rodents were trapped during June–October during 1988–2019. The trapping site was situated at ca. 525 m a.s.l. in the boreal zone at 64.40° N, 13.81–13.82° E, 444 km north-northeast of the most northern part of the study area in southeastern Norway (Fig. 1). Some years had low trapping effort, and five years (1992–1994, 1996 and 2002) with only 50–108 trap nights were excluded from the analyses. The remaining years (*n* = 27) had 192–595 trap nights per year. At Vindeln, rodents were trapped in spring and autumn during 1971–2020. The trapping site was situated at ca. 200 m a.s.l. in the boreal zone at ca. 64.20° N, 19.71° E, 595 km northeast of the most northeastern part of the study area in Oslo and Akershus (Fig. 1). At Ammarnäs, rodents were trapped in spring and autumn during 1995–2020, except during 1999–2000. The trapping site was situated at ca. 400 m a.s.l. in the boreal zone at ca. 65.96° N, 16.21° E, 647 km north-northeast of the most northern part of the study area in Oslo and Akershus (Fig. 1). At Dividalen, rodents were trapped in autumn during 1993–2020. The trapping site was situated at ca. 400–800 m a.s.l. in the boreal and alpine zone at ca. 68.70° N, 19.79° E, 989 km north-northeast of the most northern part of the study area in Oslo and Akershus (Fig. 1).

For all four sites, we used the trapping index for all microtine rodents combined, including bank vole, red-backed vole (*Myodes rutilus*), grey-sided vole (*Myodes rufocanus*), field vole, tundra vole, wood lemming (*Myopus schisticolor*) and Norway lemming (*Lemmus lemmus*). For Vindeln and Ammarnäs we only used autumn data (August–September). For details of trapping methods, see Sørensen et al. (2020), Ecke and Hörnfeldt (2021) and Framstad (2021).

At Vang, Innlandet county (Hedmark county prior to 2020), microtine rodents were trapped in late September–early October during 1981–2020 at the same site each year. The trapping site was situated at 550–600 m a.s.l. in the boreal zone at 60.94° N, 11.14° E. The site was 38 km north of the most northern part of Akershus (Fig. 1). A microtine rodent trapping index was based on all microtine species (bank vole, field vole, tundra vole and wood lemming) trapped.

At Ås (Akershus county) rodents were trapped in October during 1993–2019 at the same site each year. The trapping site was situated at 80–120 m a.s.l. in the boreo-nemoral zone at 59.69° N, 10.77° E (Fig. 1). Trapping indices based on bank vole and wood mouse (*Apodemus sylvaticus*), separately, were used. Whereas populations of the different microtine species at any site in boreal areas fluctuate in close temporal synchrony, i.e. they usually have their peak phase in the same year, and their low phase in the same year, justifying using a common trapping index for all microtine species, populations of bank vole and wood mouse at Ås, situated in the boreo-nemoral zone, are not temporally correlated, justifying using separate trapping indices for bank vole and wood mouse.

Numbers from Vang were used as an index of rodent abundance in the boreal forest region, whereas numbers from Ås were used as an index of rodent abundance in the lowlands. Online Resource 1 gives further details of trapping methods at Vang and Ås.

### Statistical analyses

The relationships between the number of owls recorded per year and rodent indices from Vang, Ås, Lierne, Vindeln, Ammarnäs and Dividalen were analysed with zero-inflated Poisson regression because there were no records of hawk owls in 22 out of 41 years of data. Rodent data were missing from some years for some sites, so that sample size was < 41 years for some sites (Vang: 40 years; Ås: 27 years; Lierne: 32 years; Vindeln: 41 years; Ammarnäs: 24 years; Dividalen: 28 years). To test whether irruptions occurred in years with low rodent abundance in northern areas, we related owl numbers to rodent indices from the same year (year *x*). To test whether irruptions were preceded by potentially high reproduction, we related owl numbers in year x to rodent indices in the two years before (year *x*-1 and year *x*-2). Rodent indices did not show long-term temporal trends (*P* = 0.14–0.94; except *P* = 0.04 for Dividalen due to two high indices at the end of the time series), so indices were not detrended. Spatial synchrony between total microtine indices across the six sites were analysed with Pearson correlations between all pairs of sites.

The relationships between irruption size and rodent indices were also analysed separately for the two subareas of the study area (see above), and restricted to the period 2011–2020 because of relatively few hawk owl records for earlier years when splitting the data into two subareas. Because there was only two years with no records of hawk owls during this 10-year period, analyses were conducted with Poisson regression without zero-inflation. Analyses of data separated into two subareas showed qualitatively similar results to the analyses based on the whole study area (Online Resource 1, Tables S5–6). Thus, the main text reports the results of analyses based on hawk owl records for the whole study area.

The relative importance of push and pull factors (i.e. rodent abundance in northern areas vs. rodent abundance in southern areas) was first assessed by the generalized *r*^2^-values from single factor zero-inflated Poisson regression models. For the subset of years for which all sites had data on rodent abundance, we also compared the AIC_c_-values of the single factor zero-inflated Poisson regression models. Second, the relative importance of push and pull factors was compared with zero-inflated Poisson regression models where both total microtine rodent indices from northern areas and the wood mouse index from Ås were predictors simultaneously. Predictors were centred (mean = 0) and scaled (sum of squares = 1). From these analyses we report the Wald χ^2^-values and the coefficient estimates for each predictor.

Relationships between elevation and habitat of hawk owls and rodent indices from Vang and Ås were first analysed with Pearson correlations where each year was one data point, and for each year the mean elevation and the proportion of the owls that were in boreal forest were calculated. Sample size included years with at least four hawk owl records (*n* = 10 years). Second, the relationship between elevation of hawk owls and rodent abundance was conducted using restricted maximum likelihood GLMM analysis with Gaussian error distribution. Each hawk owl record was used as the sampling unit. Year was included as a random effect because all records from the same year were assigned the same rodent abundance. Statistical analyses were conducted in JMP Pro version 15 (SAS 2019).

## Results

### Irruptions 1980–2020

**Fig. 2:**
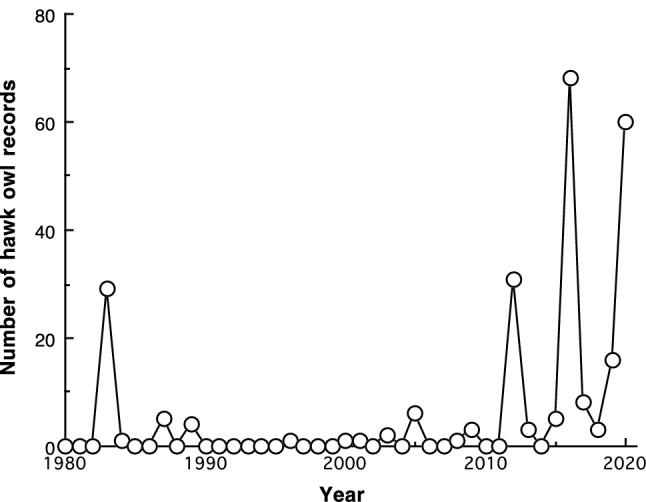
Number of Northern hawk owls (*Surnia ulula*) recorded in Oslo and Akershus, southeastern Norway, during the period 1980–2020. For each year, the number includes records from August–December and records from January–February the following year. Thus, e.g. 2020 includes records from the period August 2020–February 2021

During the period 1980–2020, there were four large irruptions: 1983, 2012, 2016 and 2020 (Fig. 2). The number of records of hawk owls was low in particular during the period 1990–2005. The number of hawk owls recorded in the northwestern and southeastern parts of the study area were positively correlated (1980–2020: *r* = 0.91, *n* = 41 years, *P* < 0.001; 2011–2020: *r* = 0.89, *n* = 10 years, *P* < 0.001).

### Push factor: irruption size in relation to rodent abundance in the north

**Fig. 3:**
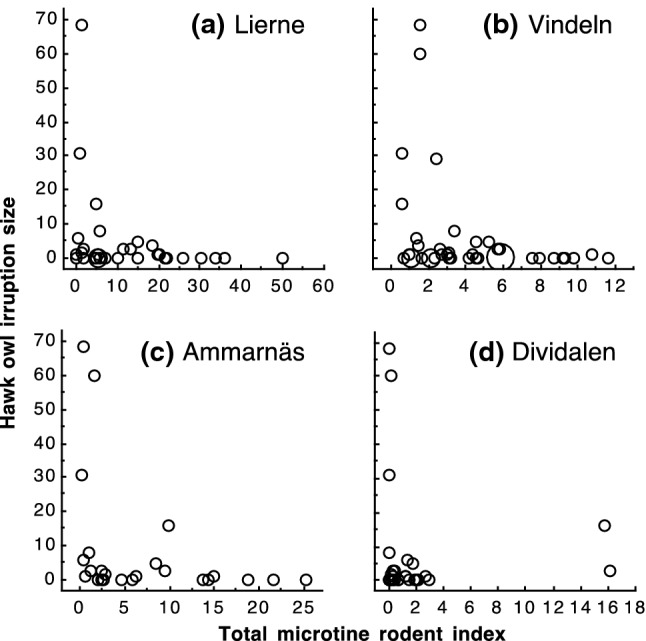
Northern hawk owl (*Surnia ulula*) irruption size (number of individuals observed per year in Oslo and Akershus, southeastern Norway) in relation to total microtine index in northern sites. **a** Lierne (*n* = 27 years), **b** Vindeln (*n* = 41 years), **c** Ammarnäs (*n* = 24 years), and **d** Dividalen (*n* = 28 years)

Irruption size (the number of hawk owls recorded in each year) was significantly negatively related to rodent abundance at northern sites that may represent source areas of irruptions (Table 1, Fig. 3). Thus, 84–90% of all hawk owl individuals were recorded in southeastern Norway in years with below median microtine rodent index at the northern sites: 136 out of 153 individuals (89%) based on data from Lierne, 222 out of 248 individuals (90%) based on data from Vindeln, 182 out of 208 individuals (88%) based on data from Ammarnäs, and 176 out of 209 individuals (84%) based on data from Dividalen. Sample sizes vary because of different lengths of trapping series at each site.

**Table 1:** Relationships between Northern hawk owl (*Surnia ulula*) irruption size (number of individuals observed per year in Oslo and Akershus, southeastern Norway) and indices of rodent abundance in the same year

	Irruption size^1^			
Rodent index	*n*	Estimate	SE	*p*
Southeastern Norway				
Vang (boreal forest)	40	– 0.028	0.011	0.013
Ås (lowlands)				
Bank vole	27	– 0.089	0.103	0.39
Wood mouse	27	1.223	0.111	< 0.001
Northern sites				
Lierne	27	– 0.132	0.018	< 0.001
Vindeln	41	– 0.479	0.049	< 0.001
Ammarnäs	24	– 0.192	0.026	< 0.001
Dividalen	28	– 0.060	0.019	0.002

^1^Zero-inflated poisson regression

Rodent abundance indices were from two sites in southeastern Norway (Vang and Ås) and four northern sites (Lierne, Vindeln, Ammarnäs, Dividalen). For all analyses except Ås, the rodent index is the total microtine rodent abundance

Additionally, irruption size was positively related to rodent abundance at the northern sites 1–2 years before irruptions (Table 2). For Lierne and Vindeln there were significant positive relationships only with rodent abundance 2 years before irruptions, but for the two northernmost sites, Ammarnäs and Dividalen, there were significant positive relationships with rodent abundance both 1 and 2 years before irruptions (Table 2).

**Table 2:** Relationships between Northern hawk owl (*Surnia ulula*) irruption size (number of individuals observed per year in Oslo and Akershus, southeastern Norway) and indices of total microtine rodent abundance in two years preceding irruptions in potential source areas (x = irruption year)

Microtine rodent index	Irruption size^1^			
	*n*	Estimate	SE	*P*
Lierne				
Year x-1	27	− 0.006	0.005	0.24
Year x-2	26	0.018	0.005	< 0.001
Vindeln				
Year x-1	40	− 0.068	0.024	0.004
Year x-2	39	0.135	0.021	< 0.001
Ammarnäs				
Year x-1	23	0.036	0.008	< 0.001
Year x-2	22	0.028	0.008	< 0.001
Dividalen				
Year x-1	27	0.093	0.009	< 0.001
Year x-2	26	0.128	0.010	< 0.001

^1^Zero-inflated poisson regression

Rodent abundance indices were from four northern sites (Lierne, Vindeln, Ammarnäs, Dividalen)

### Pull factor: irruption size in relation to rodent abundance in southeastern Norway

**Fig. 4:**
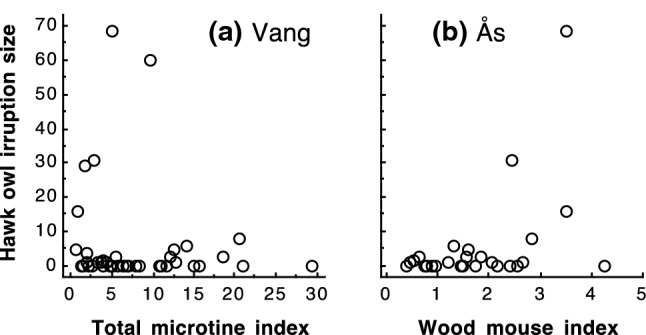
Northern hawk owl (*Surnia ulula*) irruption size (number of individuals observed per year in Oslo and Akershus, southeastern Norway) in relation to **a** total microtine index in boreal forest at Vang (*n* = 40 years), and **b** wood mouse index at Ås in the lowlands (*n* = 27 years)

At the local scale in southeastern Norway, irruption size was significantly negatively related to total microtine rodent abundance at Vang in boreal forest (Table 1, Fig. 4). Thus, 65% of all hawk owl individuals (162 out of 248) were recorded in years with below median total microtine rodent index at Vang. On the other hand, irruption size was positively related to wood mouse abundance at Ås in the lowlands (Table 1, Fig. 4). Based on hawk owls recorded in years when there was data on rodent abundance at Ås, 89% of all hawk owls were recorded in years with above median wood mouse numbers at Ås (133 out of 149 individuals).

Despite these opposing patterns, total microtine rodent abundance at Vang was not negatively correlated to wood mouse abundance at Ås (*r* = 0.03, *n* = 27 years, *P* = 0.88). Furthermore, analyses indicated that wood mouse abundance at Ås was the best predictor of irruption size when taking both total microtine abundance at Vang and wood mouse abundance at Ås into account (zero-inflated Poisson regression with centred and scaled predictors; total microtines Vang: estimate = − 0.29, SE = 0.53, *P* = 0.58, wood mouse Ås: estimate = 6.30, SE = 0.60, *P* < 0.001).

### Spatial synchrony of rodent abundance

Analyses indicated that total microtine indices were synchronous particularly among three of the northern sites (Lierne, Vindeln and Ammarnäs, Online Resource 1, Table S7). The boreal forest site in the south (Vang) was synchronous with Vindeln, and to some degree with Lierne. Otherwise, the northernmost site (Dividalen) and the lowland site in the south (Ås, bank vole only) were not synchronous with any other sites (Online Resource 1, Table S7). The wood mouse index from Ås was negatively, but weakly, correlated with total microtine indices in three of the northern areas (Lierne, Vindeln and Ammarnäs; *P* = 0.26–0.89), but weakly positively related to the index in Dividalen (*P* = 0.16).

### Relative importance of push and pull factors

In single factor zero-inflated Poisson regression models the wood mouse index from Ås in the lowlands in the south explained irruption size at least as well (generalized *r*^2^ = 0.998, *n* = 27 years) as the microtine rodent indices from northern areas (Lierne: *r*^2^ = 0.913, *n* = 27 years; Vindeln: *r*^2^ = 0.959, *n* = 41 years; Ammarnäs: *r*^2^ = 0.952, *n* = 24 years; Dividalen: *r*^2^ = 0.373, *n* = 28 years). For the 21 years where all sites had data, the wood mouse index from Ås explained irruption size better (generalized *r*^2^ = 0.999, AIC_c_ = 155.93) than the microtine rodent indices from northern areas (Lierne: *r*^2^ = 0.942, AIC_c_ = 251.17; Vindeln: *r*^2^ = 0.980, AIC_c_ = 228.45; Ammarnäs: *r*^2^ = 0.871, AIC_c_ = 267.95; Dividalen: *r*^2^ = 0.239, AIC_c_ = 305.27). The null model (only intercept) had AIC_c_ = 308.24. Zero-inflated Poisson regression models of irruption size with both total microtine rodent indices from northern areas and wood mouse index from Ås as predictors (main push and pull factors), showed that both were of relatively similar magnitude based on centred and scaled coefficient estimates (Table 3).

**Table 3:** Relative importance of push (northern rodent abundance indices) and pull (wood mouse index at Ås) factors for explaining Northern hawk owl (*Surnia ulula*) irruption size (number of individuals observed per year in Oslo and Akershus, southeastern Norway)

Model	Irruption size^1^			
	*n*	Coefficient estimate	SE	Wald χ^2^
Model 1				
Microtines Lierne	23	− 12.25	2.22	30.53
Wood mouse Ås	23	4.50	0.45	100.93
Model 2				
Microtines Vindeln	27	− 5.45	1.10	24.72
Wood mouse Ås	27	4.89	0.61	65.04
Model 3				
Microtines Ammarnäs	23	− 5.35	0.87	37.81
Wood mouse Ås	23	4.95	0.44	125.30
Model 4				
Microtines Dividalen	27	− 1.70	0.35	23.55
Wood mouse Ås	27	7.16	0.61	136.14

^1^Zero-inflated poisson regression with centred and scaled predictors

Four models with rodent abundance indices from each of the four northern areas (Lierne, Vindeln, Ammarnäs, Dividalen) are shown

### Elevation and habitat

**Fig. 5:**
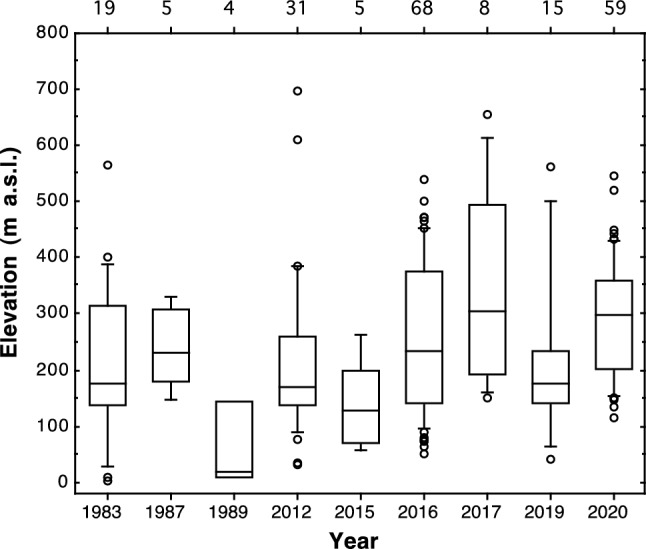
Elevation of Northern hawk owl (*Surnia ulula*) records during irruptions to Oslo and Akershus, southeastern Norway. Years with at least four records are shown. For each year, the number includes records from August–December and records from January–February the following year. Thus, e.g. 2020 includes records from the period August 2020–February 2021. Boxes indicate 25th, 50th and 75th percentiles, whiskers indicate 10th and 90th percentiles. Numbers on top of graph indicate yearly sample sizes (number of hawk owl records with information on elevation)

Overall, elevation of hawk owl records ranged from 4 to 698 m a.s.l. (mean = 243, median = 202; *n* = 233). Elevation of records during years with at least four records showed substantial variation with most birds observed in the lowlands in some years and in the hills in other years (Fig. 5). For the 6 years with at least eight records, elevation showed significant variation between years (ANOVA: *F* = 2.89, *df* = 5, *P* = 0.015).

Overall, 60% of hawk owl records were from boreal forest and 40% were from farmland (*n* = 237). There was significant variation between years in the distribution of records in relation to habitat (six years with at least eight records: χ^2^ = 37.44, *df* = 5, *P* < 0.001). Hawk owls were predominantly in boreal forest in 2016 (63%), 2017 (88%) and 2020 (87%), whereas the majority was in farmland in 1983 (63%), 2012 (65%), and 2019 (60%). Mean elevation within years and proportion of the hawk owls recorded in boreal forest were significantly correlated (years with at least four records with information on both elevation and habitat: *r* = 0.84, *n* = 9 years, *P* = 0.005). Mean elevation and habitat use were not significantly related to irruption size (mean elevation: *r* = 0.39, *n* = 9 years, *P* = 0.30; proportion recorded in boreal forest: *r* = 0.28, *n* = 10 years, *P* = 0.44).

### Rodent abundance and elevation/habitat

The proportion of the hawk owls that were recorded in boreal forest was positively correlated with the total microtine rodent abundance in boreal forest at Vang (Table 4). On the other hand, the relative distribution of owls between boreal forest and farmland was not related to bank vole or wood mouse abundance at Ås in the lowlands.

**Table 4:** Relationships between rodent abundance and elevation/habitat of records of Northern hawk owls (*Surnia ulula*) during irruptions to Oslo and Akershus, southeastern Norway. Year-based analyses used proportion of hawk owls recorded in boreal forest (as opposed to in farmland), and mean elevation of hawk owl records for years with at least four records (*n* = 10 years). Individual-based analyses used all hawk owl records with information on elevation (*n* = 233 individuals)

	Use of boreal forest		Mean elevation		Elevation		
	Year-based analyses^1^		Year-based analyses^1^		Individual-based analyses^2^		
Rodent index	*r*	*P*	*r*	*P*	Estimate	SE	*P*
Vang (boreal forest in hills)							
Total microtines	0.79	0.007	0.77	0.014	5.79	2.50	0.039
Ås (lowlands)							
Bank vole	− 0.43	0.40	− 0.71	0.18	− 32.00	24.37	0.22
Wood mouse	0.28	0.60	0.50	0.39	29.33	60.02	0.89

^1^Pearson correlations

^2^GLMM-analyses with year as random effect

Rodent abundance indices were available from Vang during 1981–2020 (providing 10 years of data for habitat, 9 years for elevation, 233 hawk owl individuals), and from Ås during 1993–2019 (6 years of data for habitat, 5 years for elevation, 145 hawk owl individuals)

**Fig. 6:**
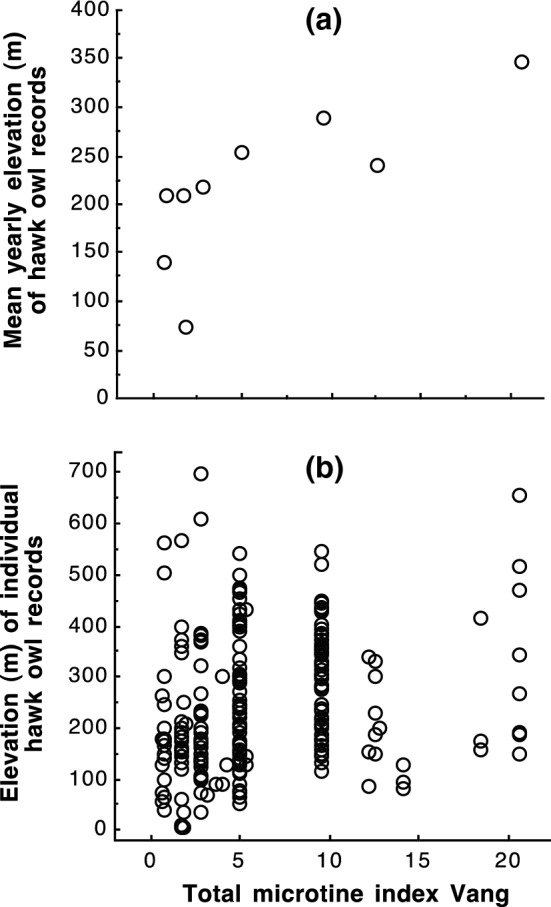
Elevation (m a.s.l.) of Northern hawk owl (*Surnia ulula*) records in Oslo and Akershus, southeastern Norway, in relation to total microtine index in boreal forest at Vang. **a** Mean yearly elevation of records for years with at least four records (*n* = 9 years), and **b** elevation of individual records (*n* = 233 hawk owl records)

Similarly, the yearly mean elevation of hawk owl records was positively correlated with the abundance of rodents in boreal forest at Vang (Table 4; Fig. 6). The yearly mean elevation of hawk owl records was not related to rodent abundance indices at Ås in the lowlands, but note that these analyses were based on only five years of data. The individual-based analyses also indicated that elevation of records was positively related to the total microtine index at Vang, but not rodent indices at Ås (Table 4; Fig. 6).

Based on hawk owls recorded in years when there was data on rodent abundance at Ås in the lowlands, 48% (71 out of 149 individuals) were recorded in the lowlands. Among the individuals recorded in the lowlands, 87% (62 out of 71 individuals) were recorded in a year with above median wood mouse abundance.

## Discussion

### Irruptions

Hawk owl irruptions to southeastern Norway occurred at irregular intervals. After the large irruption in 1983, hawk owls were present in particular at higher elevations in eastern Norway during some years (Sonerud 1997). Hawk owls were thereafter more or less absent for many years, in particular during 1990–2005. However, from 2006 hawk owls have become much more common again, with three large irruption years (2012, 2016 and 2020), and increasing number of observations even in years between the irruption peaks. This suggests that the population size and distribution range of hawk owls have increased recently. One possible reason may be the return of more regular rodent cycles with larger peaks from ca. 2005–2010 after a period of dampened cycles starting around 1990 (Brommer et al. 2010; Wegge and Rolstad 2018; Selås et al. 2021; Sonerud 2022).

Another possibility is that the larger number of hawk owl records from about 2010 could be due to increased observation effort and reporting by birders. However, the 15-fold increase in hawk owl reports from 2001–2010 to 2011–2020 was not matched by a similar increase in number of bird reports in general on www.artsobservasjoner.no, where the increase was only threefold (from on average 348,682 to 1,119,488 bird reports per year). Note that our data set also included other sources of information than www.artsobservasjoner and should therefore be less affected by a temporal trend in number of reports than www.artsobservasjoner.no which was launched in 2006.

### Importance of push factor

Hawk owl irruptions are thought to be triggered when rodent numbers in northern areas crash after a population peak, forcing the owls to emigrate (push hypothesis). The four-year intervals between the most recent irruption peaks in the study area point to rodent cycles as an important cause because rodent cycles in Fennoscandia typically have 3–5 year intervals. This was confirmed in our analyses of how irruption size was related to rodent abundance at four northern sites representing potential source areas for irruptions. Irruption size was significantly negatively related to rodent abundance at the northern sites in all analyses, and 84–90% of all hawk owl individuals in southeastern Norway were recorded in years with below median microtine rodent index in northern sites. These results support the push hypothesis.

Furthermore, we found that irruption size was positively related to rodent abundance in northern areas 1–2 years before irruptions. This suggests that there may have been a high breeding output for hawk owls in the years preceding the irruptions allowing population size to build up. Irruptions may then have been triggered by the interaction between large population size and low prey availability. One would expect that irruptions would in particular be triggered if there was a good breeding season just one year before rodent abundance declined to a low level. This was found only for Ammarnäs and Dividalen, the two most northern sites. One interpretation of this pattern could be that the main source areas for irruptions are in the most northern areas of Fennoscandia.

### Importance of pull factor

Irruption size was negatively related to total microtine rodent abundance in boreal forest at Vang in southeastern Norway, and 65% of all hawk owl individuals were recorded in years with below median total microtine rodent index at Vang. Thus, there was no evidence that hawk owls were pulled to southeastern Norway by high rodent abundance in the boreal forest, where microtine rodents dominate and wood mouse hardly occurs.

However, irruption size was positively related to wood mouse abundance in the lowlands of southeastern Norway, as indicated by trapping indices at Ås. Thus, 89% of all hawk owls were recorded in years with above median wood mouse numbers at Ås. On the other hand, the high availability of wood mouse was not 'discovered' by all hawk owls. Overall, only 40% of all hawk owls were recorded in farmland in the lowlands, but among these 87% were recorded in years with above median wood mouse abundance. This suggests that high availability of wood mouse may have worked as a pull factor for at least part of the hawk owl individuals involved in irruptions.

The correlation between irruption size and the wood mouse suggests that hawk owls prey on wood mouse. However, although hawk owls have been documented to take wood mouse, they apparently occur as prey only in low numbers (Mikkola 1971). Furthermore, whereas hawk owls are mainly diurnal, the wood mouse is mainly nocturnal (Greenwood 1978; Wolton 1983; Flowerdew 2000). However, almost all data on the diet of hawk owls are from breeding areas where the wood mouse is rare or absent. Thus, it is possible that the winter diet of hawk owls in the lowlands may differ from their diet in boreal forests during the breeding season. Also, although mainly diurnal, it is possible that hawk owls may be able to hunt during nights and supplement their diet with wood mice, or that some wood mice may be taken during daytime because this species is active to some degree during daytime in the study area at Ås (G.A. Sonerud, personal observations). Thus, the results of this study suggest that the wood mouse may be an alternative prey for hawk owls during winter in the lowlands, but this needs to be confirmed through more detailed studies of their diet during these circumstances.

The hawk owls that stayed in boreal forest during irruptions did so in particular in years with higher microtine rodent abundance, suggesting that prey abundance was large enough to survive there. Thus, it appears that the hawk owls stayed in boreal forests as long as prey were available, but moved to farmland areas at lower elevations if prey were scarce. This suggests that owls were forced to use lowlands with open farmland habitat because of lack of prey in boreal forest. Given that hawk owls have boreal forests as their main habitat, one may speculate that use of other habitats may be suboptimal, perhaps because of predation risk from the Northern goshawk (*Accipiter gentilis*) which is more common in the lowlands (Bergo 1994).

### Relative importance of push and pull factors

Analyses that included both push and pull factors (microtine abundance in northern areas and wood mouse abundance in the study area, respectively) suggested that their relative magnitude was quite similar. Single factor analyses even suggested that irruptions were explained better by wood mouse abundance at Ås than microtine abundance in northern areas. Also, analyses of local factors indicated that wood mouse abundance at Ås was a better predictor of irruption size than total microtine abundance at Vang despite no significant relationship between total microtine abundance at Vang and wood mouse abundance at Ås. There was data on wood mouse abundance for three of the five largest irruptions, and in all of these years total microtine abundance at Vang was below median and wood mouse abundance at Ås was above median.

### How does a pull factor work?

Hawk owls obviously have no direct knowledge of prey availability in distant areas. The individual decisions to move long distances that underly an irruption may, however, have been shaped by natural selection to become an adaptive strategy. If prey availability is predictably negatively correlated between source and destination areas, hawk owls may have been selected to have a behavioural rule that 'if prey are scarce in northern areas, move southwards until better conditions are encountered', and this may on average help individuals to find an area where they can survive until rodent abundance in northern areas has started to increase again. Spatial asynchrony in prey availability for hawk owls may in turn be related to predictable climatic asynchrony similar to how climatic dipoles at larger scales may affect plants and animals (Strong et al. 2015; Zuckerberg et al. 2020).

The direction of migration during irruptions might be thought to be random, although in Fennoscandia obviously without a northern component. However, during the 2016 irruption hawk owls from northern Fennoscandia moved mainly towards south-southwest to southern Norway and Sweden, and to a much smaller degree towards south-southeast, because there were relatively few observations in southern Finland and further east (Dale 2017). This may suggest that hawk owls migrate in directions that are particularly profitable.

In some years hawk owls would encounter excellent conditions in the destination areas leading to high survival and even breeding in southern boreal forests, but in other years rodents have extended low phases leading to poor survival whichever habitat hawk owls select in the destination areas. Survival in destination areas may also be affected by density-dependence (McCabe et al. 2021). Yet, pull factors that affect irruptive behaviour contrast with older ideas that irruptions often lead to high mortality (e.g. suicidal Norway lemming movements; see Chitty 1996 for a critical review), and therefore represent a best-of-a-bad-job strategy. Our findings support the idea that irruptive behaviour may be an optimal strategy that likely ensures survival for many hawk owls even after their prey populations in northern areas have crashed. Irruptive behaviour is thereby shaped by spatial and temporal variation in rodent abundance creating push and pull dynamics that affect individual migration decisions.

## Supplementary Information

Below is the link to the electronic supplementary material.

Supplementary file1 (DOCX 27 KB)

## Data Availability

The datasets used and analysed during the current study are available from the corresponding author upon request.

## Code availability

We have used standardised modelling so no code is provided.
