# Supplementary material for: Hawk owl irruptions: spatial and temporal variation in rodent abundance drive push and pull dynamics
Source: Oecologia. 2022 Nov 19;201(1):31–43. doi: 10.1007/s00442-022-05283-9 (PMC9813069; doi:10.1007/s00442-022-05283-9)
Supplement: Supplementary file 1 — Supplementary file1 (DOCX 27 KB) [file 442_2022_5283_MOESM1_ESM.docx]

**Supplementary material to:**

Hawk owl irruptions: spatial and temporal variation in rodent abundance drive push and pull dynamics

Oecologia

Svein Dale and Geir A. Sonerud

Faculty of Environmental Sciences and Natural Resource Management, Norwegian University of Life Sciences, P.O. Box 5003, NO-1432 Ås, Norway.

Corresponding author: Svein Dale, e-mail: svein.dale@nmbu.no.

**Supplementary methods**

**Rodent trapping at Vang**

At Vang, Innlandet county (Hedmark county prior to 2020), microtine rodents were trapped in late September – early October during 1981–2020 at the same site each year. In each trapping session ca. 300 wooden snap traps (brand Rapp) baited with a piece of yarn soaked with melted cocoa fat (brand Delfia) were put out and checked each morning for 4 days. The traps were set ca. 5 m apart in seven separate lines > 160 m apart within an area of ca. 40 ha. The lines were kept the same through all 40 trapping years while the forest cover changed. Most traps were in a clear-cut at the start and in middle-aged forest at the end, while the others were in old forest at the start and in plantations or young forest at the end; see Sonerud (1986, 1988) for a description of the trapping area in the first years of the trapping. There were 1,000–1,184 trap nights per year. A microtine rodent trapping index was calculated as number of animals of all microtine species (bank vole, field vole, tundra vole and wood lemming) trapped per 100 trap nights.

**Rodent trapping at Ås**

At Ås, Viken county (Akershus county prior to 2020) rodents were trapped in October during 1993–2019 at the same sites each year. The traps were set in four habitat patches, which at the start in 1993 were an old spruce forest, a fresh clear-cut in spruce forest, a fresh forest burn, and a lush broadleaf forest, respectively. In each habitat, five 15 m x 15 m Small Quadrats (SQ; see Myllymäki et al. 1971) were permanently established, in total 20 SQs within ca. 100 ha. The nearest neighbor distance between the SQs within a habitat ranged ca. 50–100 m, and the nearest neighbor distance between the habitat patches ranged ca. 300–1,500 m. In each corner of each quadrat, 3 wooden snap traps (brand Rapp) baited with a piece of yarn soaked with soya oil (1993–2002) or melted cocoa fat (2003–2019) were put out and checked each morning for two days. Thus, there were 480 trap nights per year. Parts of the old forest were logged on several occasions during the study, so some SQs had to be moved several times to remain in old forest habitat throughout the study. In the other habitats, the SQs were at the same sites throughout the study. For bank vole and wood mouse (*Apodemus sylvaticus*) separately, trapping indices were calculated as number of animals trapped per SQ.

**References**

Myllymäki A, Paasikallio A, Pankakoski E, Kanervo V (1971) Removal experiments on small quadrats as a means of rapid assessment of the abundance of small mammals. Ann Zool Fenn 8: 177–185. <https://www.jstor.org/stable/23731835>

Sonerud GA (1986) Effect of snow cover on seasonal changes in diet, habitat, and regional distribution of raptors that prey on small mammals in boreal zones of Fennoscandia. Holarctic Ecol 9: 33–47. <https://doi.org/10.1111/j.1600-0587.1986.tb01189.x>

Sonerud GA (1988) What causes extended lows in microtine cycles? Analysis of fluctuations in sympatric shrew and microtine population in Fennoscandia. Oecologia 76: 37-42. <https://doi.org/10.1007/BF00379597>

**Table S1** Relationships between Northern hawk owl (*Surnia ulula*) irruption size (number of individuals observed per year in Oslo and Akershus, southeastern Norway **adjusted for yearly increase in bird reporting volume**^1^) and indices of rodent abundance in the same year. Rodent abundance indices were from two sites in southeastern Norway (Vang and Ås) and four northern sites (Lierne, Vindeln, Ammarnäs, Dividalen). For all analyses except Ås, the rodent index is the total microtine rodent abundance

______________________________________________________________________

Irruption size^2^

Rodent index *n* Estimate SE *P*

______________________________________________________________________

**Southeastern Norway**

Vang (boreal forest) 40 -0.066 0.010 < 0.001

Ås (lowlands)

Bank vole 27 -0.090 0.094 0.34

Wood mouse 27 1.050 0.095 < 0.001

**Northern sites**

Lierne 27 -0.102 0.015 < 0.001

Vindeln 41 -0.299 0.033 < 0.001

Ammarnäs 24 -0.195 0.025 < 0.001

Dividalen 28 -0.068 0.019 < 0.001

______________________________________________________________________

^1^ Temporal changes in bird reporting volume was based on data from www.artsobservasjoner.no (launched in 2006). For the period 2006–2020 the yearly total number of bird reports increased with 0.043 per year relative to an index of 1 in 2006. For the period 1980–2005 the yearly total number of bird reports increased with 0.113 per year relative to an index of 1 in 1980. The number of owls was therefore, for example, adjusted from 29 to 137 for the 1983–84 irruption

^2^ Zero-inflated Poisson regression

**Table S2** Relationships between Northern hawk owl (*Surnia ulula*) irruption size (number of individuals observed per year in Oslo and Akershus, southeastern Norway) and indices of rodent abundance in the same year. Rodent abundance indices were from two sites in southeastern Norway (Vang and Ås) and four northern sites (Lierne, Vindeln, Ammarnäs, Dividalen). For all analyses except Ås, the rodent index is the total microtine rodent abundance. **Analyses were restricted to the period 2011–2020**

______________________________________________________________________

Irruption size^1^

Rodent index *n* Estimate SE *P*

______________________________________________________________________

**Southeastern Norway**

Vang (boreal forest) 10 -0.062 0.010 < 0.001

Ås (lowlands)

Bank vole 9 -0.180 0.105 0.087

Wood mouse 9 1.213 0.128 < 0.001

**Northern sites**

Lierne 9 -0.236 0.027 < 0.001

Vindeln 10 -0.475 0.045 < 0.001

Ammarnäs 10 -0.215 0.025 < 0.001

Dividalen 10 -0.094 0.020 < 0.001

______________________________________________________________________

^1^ Poisson regression

**Table S3** Relationships between Northern hawk owl (*Surnia ulula*) irruption size (number of individuals observed per year in Oslo and Akershus, southeastern Norway **adjusted for yearly increase in bird reporting volume**, see Table S1 for further information) and indices of total microtine rodent abundance in two years preceding irruptions in potential source areas (x = irruption year). Rodent abundance indices were from four northern sites (Lierne, Vindeln, Ammarnäs, Dividalen)

______________________________________________________________________

Microtine Irruption size^1^

rodent index *n* Estimate SE *P*

______________________________________________________________________

Lierne

Year x-1 27 -0.0002 0.004 0.96

Year x-2 26 0.019 0.004 < 0.001

Vindeln

Year x-1 40 -0.125 0.020 < 0.001

Year x-2 39 0.181 0.016 < 0.001

Ammarnäs

Year x-1 23 0.040 0.007 < 0.001

Year x-2 22 0.030 0.008 < 0.001

Dividalen

Year x-1 27 0.081 0.009 < 0.001

Year x-2 26 0.117 0.009 < 0.001

______________________________________________________________________

^1^ Zero-inflated Poisson regression

**Table S4** Relationships between Northern hawk owl (*Surnia ulula*) irruption size (number of individuals observed per year in Oslo and Akershus, southeastern Norway) and indices of total microtine rodent abundance in two years preceding irruptions in potential source areas (x = irruption year). Rodent abundance indices were from four northern sites (Lierne, Vindeln, Ammarnäs, Dividalen). **Analyses were restricted to the period 2011–2020**

______________________________________________________________________

Microtine Irruption size^1^

rodent index *n* Estimate SE *P*

______________________________________________________________________

Lierne

Year x-1 10 -0.002 0.007 0.74

Year x-2 10 0.053 0.006 < 0.001

Vindeln

Year x-1 10 -0.125 0.026 < 0.001

Year x-2 10 0.316 0.031 < 0.001

Ammarnäs

Year x-1 10 0.033 0.008 < 0.001

Year x-2 10 0.045 0.008 < 0.001

Dividalen

Year x-1 10 0.067 0.010 < 0.001

Year x-2 10 0.106 0.010 < 0.001

______________________________________________________________________

^1^ Poisson regression

**Table S5** Relationships between Northern hawk owl (*Surnia ulula*) irruption size (number of individuals observed per year **in two subareas** of Oslo and Akershus, southeastern Norway) and indices of rodent abundance in the same year. Rodent abundance indices were from two sites in southeastern Norway (Vang and Ås) and four northern sites (Lierne, Vindeln, Ammarnäs, Dividalen). For all analyses except Ås, the rodent index is the total microtine rodent abundance. Analyses were restricted to the period 2011–2020

______________________________________________________________________

Irruption size^1^

Rodent index *n* Estimate SE *P*

______________________________________________________________________

Northwestern subarea

**Southeastern Norway**

Vang (boreal forest) 10 -0.055 0.013 < 0.001

Ås (lowlands)

Bank vole 9 -0.164 0.142 0.25

Wood mouse 9 1.172 0.171 < 0.001

**Northern sites**

Lierne 9 -0.208 0.033 < 0.001

Vindeln 10 -0.463 0.058 < 0.001

Ammarnäs 10 -0.182 0.029 < 0.001

Dividalen 10 -0.060 0.021 0.004

Southeastern subarea

**Southeastern Norway**

Vang (boreal forest) 10 -0.073 0.017 < 0.001

Ås (lowlands)

Bank vole 9 -0.199 0.157 0.20

Wood mouse 9 1.266 0.195 < 0.001

**Northern sites**

Lierne 9 -0.279 0.047 < 0.001

Vindeln 10 -0.493 0.071 < 0.001

Ammarnäs 10 -0.284 0.050 < 0.001

Dividalen 10 -0.193 0.056 < 0.001

______________________________________________________________________

^1^ Poisson regression

**Table S6** Relationships between Northern hawk owl (*Surnia ulula*) irruption size (number of individuals observed per year **in two subareas** of Oslo and Akershus, southeastern Norway) and indices of total microtine rodent abundance in two years preceding irruptions in potential source areas (x = irruption year). Rodent abundance indices were from four northern sites (Lierne, Vindeln, Ammarnäs, Dividalen). Analyses were restricted to the period 2011–2020

______________________________________________________________________

Microtine Irruption size^1^

rodent index *n* Estimate SE *P*

______________________________________________________________________

Northwestern subarea

Lierne

Year x-1 10 -0.012 0.009 0.18

Year x-2 10 0.041 0.008 < 0.001

Vindeln

Year x-1 10 -0.157 0.035 < 0.001

Year x-2 10 0.265 0.040 < 0.001

Ammarnäs

Year x-1 10 0.028 0.011 0.013

Year x-2 10 0.038 0.010 < 0.001

Dividalen

Year x-1 10 0.084 0.013 < 0.001

Year x-2 10 0.116 0.013 < 0.001

Southeastern subarea

Lierne

Year x-1 10 0.011 0.010 0.28

Year x-2 10 0.071 0.010 < 0.001

Vindeln

Year x-1 10 -0.082 0.040 0.041

Year x-2 10 0.393 0.051 < 0.001

Ammarnäs

Year x-1 10 0.040 0.013 0.002

Year x-2 10 0.055 0.012 < 0.001

Dividalen

Year x-1 10 0.042 0.016 0.010

Year x-2 10 0.092 0.016 < 0.001

______________________________________________________________________

^1^ Poisson regression

**Table S7** Cross-correlations between total microtine rodent indices (only bank vole index for Ås) from six sites in Norway and Sweden. Pearson correlation coefficient (above), *P*-value (below). Significant values are shown in bold

______________________________________________________________________

Site Å Va L Vi A

______________________________________________________________________

**Southeastern Norway**

Ås (Å) - - - - -

Vang (Va) 0.26 - - - -

0.19

**Northern sites**

Lierne (L) 0.13 0.33 - - -

0.55 0.096

Vindeln (Vi) 0.11 **0.42** **0.74** - -

0.59 **0.007** **< 0.001**

Ammarnäs (A) 0.14 0.14 **0.82** **0.60** -

0.53 0.50 **< 0.001** **0.002**

Dividalen (D) 0.07 0.02 0.01 -0.04 0.23

0.71 0.92 0.95 0.83 0.29

______________________________________________________________________
